# On the Difficulty of Preserving the Vaccine Virus on Thread or Glass in Very Hot Weather

**Published:** 1801-10

**Authors:** 


Extract of a Letter from Profeffor WATERHOUSE, of Cambridge, near Bofton, Maffachufetts, to Dr. LETTSOM, of London, dated Auguft 6, 1801.

### *On the Difficulty of preserving the Vaccine Virus on Thread or Glass in very hot Weather.*

THERE is a fact of fome importance, not, I fufpect, generally known to thofe who practife the Vaccine Inoculation, even in England. It relates to the prefervation of the matter, or virus, on thread or glafs, for the purpofe of inoculation. It has been fufficiently proved, that in moderate weather, when the mercury ranged between 40° and 70° of Fahrenheit's thermometer, the Vaccine virus has retained its activity three months, and even longer. But I have lately found, that perfectly active virus, taken on cotton thread, from cafes every way fatisfactory, (proved fo by their recent fluid communicating the difeafe in perfection) will lofe its virtue when expofed ten or twelve hours to 95 or 96 degrees of Fahrenheit's fcale. It appears, that in fuch a temperature the virus or Vaccine *aura* evaporates. Ever fince the beginning of laft March, I have inoculated more or lefs almoft every day, and have not experienced a fingle difappointment when I took the limpid fluid from patient to patient; and but few inftances of failure when I had recourfe to the *thread*. But during our late very hot and very dry weather, the thread has failed in almoft every inftance; when I have fent it to practitioners in the country, enclofed in a letter, it has not in one inftance proved fuccefsful.

By

By the Abstract from the Meteorological Register * kept in this University by the Professor of Experimental Philosophy, you will see that the mercury rose almost every day for a fortnight to about 90; sometimes 96, and once 97 degrees in the shade; and that the hygrometer pointed to 40, and even to 39°. † That this great degree of heat was accompanied with an

---

* THERMOMETER and HYGROMETER, at Cambridge, from June 23, to July 16, 1801.

| Day. | Hour. | Ther. | Hygr. | Day. | Hour. | Ther. | Hygr. | Day. | Hour. | Ther. | Hygr. |
|---|---|---|---|---|---|---|---|---|---|---|---|
| June 23 | 7<br>2<br>9 | 74°<br>79<br>66 | 65°<br>57<br>63 | July 1 | 7<br>2<br>9 | 76°<br>95<br>77 | 65°<br>49<br>58 | July 9 | 7<br>2<br>9 | 71°<br>82<br>62½ | 53°<br>46<br>52 |
| 24 | 7<br>2<br>9 | 78<br>96<br>74 | 60<br>49<br>59 | 2 | 7<br>2<br>9 | 79<br>97<br>81 | 61<br>47<br>52 | 10 | 7<br>2<br>9 | 65<br>89<br>68 | 55<br>42<br>45 |
| 25 | 7<br>2<br>9 | 72<br>85<br>69 | 59<br>46<br>52 | 3 | 7<br>2<br>9 | 82<br>86<br>73 | 57<br>50<br>61 | 11 | 7<br>2<br>9 | 71<br>91<br>72 | 50<br>43<br>47 |
| 26 | 7<br>2<br>9 | 70<br>83<br>73 | 56<br>50<br>55 | 4 | 7<br>2<br>9 | 75<br>80<br>61 | 58<br>46<br>55 | 12 | 7<br>2<br>9 | 74<br>95<br>74 | 54<br>44<br>51 |
| 27 | 7<br>2<br>9 | 72½<br>92<br>74½ | 63<br>52<br>60 | 5 | 7<br>2<br>9 | 62<br>78<br>63 | 60<br>49<br>52 | 13 | 7<br>2<br>9 | 76<br>92<br>68 | 61<br>49<br>69 |
| 28 | 7<br>2<br>9 | 74<br>91<br>79 | 59<br>46<br>50 | 6 | 7<br>2<br>9 | 70<br>88½<br>68 | 52<br>39<br>46 | 14 | 7<br>2<br>9 | 73<br>81<br>64 | 68<br>57<br>65 |
| 29 | 7<br>2<br>9 | 78<br>97<br>74 | 53<br>42<br>50 | 7 | 7<br>2<br>9 | 77<br>93<br>75½ | 50<br>40<br>45 | 15 | 7<br>2<br>9 | 69<br>84<br>73½ | 66<br>48<br>55 |
| 30 | 7<br>2<br>9 | 72<br>81<br>64 | 54<br>51<br>62 | 8 | 7<br>2<br>9 | 73<br>94<br>75 | 48<br>39<br>46 | | | | |

July 3, the Thermometer shewed 95°, at 1 o'clock. From July the 4th to the 12th, both inclusive, there was no rain.

† The thermometer is Fahrenheit's, made by Champney. It is fixed on two bars without a chamber window, in the north side of the house. The hygrometer is De Luc's, made by Jones, and is *new*; it is suspended without the same window.

an extraordinary drynefs in the atmofphere was noticed by our hay-makers, there being fcarcely any appearance of dew at fun-rife for nearly a week. Finding thefe failures in the ufe of the thread, I was led to inveftigate the caufe, and am now fatisfied that it arofe from the great heat and drynefs which have reign-ed for two or three weeks paft.

If a thread dipped in the oil of lavender lofes its fragrance by an expofure for feveral hours to the degree of heat juft de-fcribed, can we wonder that the more fubtle AURA *vaccina* fhould in like manner evaporate, and the virus become effete? What tended to confirm me in this opinion was a letter from the celebrated JENNER. It is this: "I fhall juft fay a word "in proof of the *extreme delicacy* of the nature of the Vaccine "fluid, to fhow how eafily it may be diforganized. In the "early part of my practice, I ufed frequently to evaporate the "fluid by the fire upon *threads*, *glass*, or *lancets*, yet with much "caution refpecting the degree of heat; but experience has "taught me, that even this procedure frequently occafioned "an unnatural deviation from its perfect ftate, and a failure in "communicating the difeafe."

I hope not to be mifunderftood.—I can never defire more fatisfactory cafes of *Kine Pox* (as it is univerfally denominated in this region) than have occurred at this warm feafon, but they have been produced by the recent fluid, transferred from patient to patient; whereas thread, ever fo well imbued with the fluid of thefe fatisfactory cafes, and kept for a week or ten days, has loft its virtue; being, as I conceive, evaporated and exficcated by a feries of hot and dry days. It is recorded, that when the *Harmattan* blows, (which is a very hot and a very dry wind in Africa, when the backs of books crack open, and cabinet furniture fly to pieces) the Small-pox could not be communicated by inoculation.* It is probable the failure arofe from the caufe juft mentioned.

As the mercury, if I am not miftaken, very rarely rifes a-bove 84° in Britain, your phyficians could not have been led to thefe obfervations. They are, however, of fome importance, as it regards the tranfmiffion of the Vaccine virus to the hot regions of India. It is probably owing to this caufe that the Vaccine Inoculation has not profpered in the Weft Indies, while it has fucceeded at Newfoundland.

The virus I received from Dr. Haygarth, from yourfelf re-peatedly, from Mr. Creafer of Bath, from Jenner himfelf, from Mr. Ring, from Dr. Woodville, from the Vaccine Inftitution, from Dr. Pearfon, and from Mr. Kerre, have demonftrated

* Philofophical Tranfactions for 1781.

that it will *live* nearly four months. Some I received from Mr. Dunning of Plymouth, after a long and circuitous rout of five months, failed in producing the genuine puſtule; it was not, however, totally effete.

Being obliged to keep the infected thread by me, to inoculate ſuch as come to my houſe for that purpoſe, I have been in the habit of placing the phial containing the infected thread in a veſſel of cold water, in the cooleſt room in the houſe, and on a marble table. When I ſend it abroad, I encloſe the thread in a quill, corked and ſealed up, and wrapped round with thin ſheet lead, (ſuch as comes from China in tea cheſts) which preſerves it cool, and at the ſame time excludes the circumambient air; a ſample of which I ſend by this opportunity to Mr. Ring, in return for ſome active virus he has juſt ſent to me. In this way, I preſume, the Vaccine virus may be ſent to the Eaſt Indies with undiminiſhed virtue.

I wrote to Dr. Jenner, not long ſince, that I had diſcovered the Vaccine diſeaſe among our own cows; but I have lately been convinced that an unlucky youth had inoculated the cow from a human ſubject. An elderly man, who had long ſerved the public in the double capacity of inn-keeper and phyſician, was noted for his unbelief in, and oppoſition to, this new inoculation. Near him reſided a medical pupil who profeſſed a different faith, and who wiſhed to introduce the inoculation in the village; but the influence of this old Chiron prevailed, who declared his total unbelief even of the exiſtence of ſuch a diſeaſe as the Cow or Kine Pox. It was well known, that his daughters, who were his milk-maids, as well as his wife, were liable to Small-pox. This young man, therefore, ſlily inoculated one of the *doctor's* milch-cows, which ſoon conveyed the diſtemper to one of the daughters, who being diſabled from milking, another took her place; and ſhe being ſoon infected, a third aſſiſted, and, finally, the mother,* who was labouring under very heavy ſymptoms of the diſeaſe, having a number of puſtules on her hands and arms, when a gentleman of the law, who had ſeen the diſorder in his own family, and knew it, accidentally ſaw her, and wrote me an account of it; but was totally ignorant of its artificial origin, for the young man kept his ſecret till very lately. Whether this mode of converting unbelievers will prevail with us, I am unable to ſay; but this inſtance had that effect on the old gentleman, as he has been heard

* I ſuſpect that the diſorder can be conveyed from the teats of the cow to the milkers, even if they have no crack or wounds in the ſkin of their hands, ſo few milkers appear to eſcape it.

heard to declare, that if he could poſſibly believe there ever exiſted ſuch a diſorder, he ſhould be almoſt ready to think that his cows, his daughters, and his wife, had the Kine Pox, or ſomething like it.

